# Dynamics of Coral Reef Benthic Assemblages of the Abrolhos Bank, Eastern Brazil: Inferences on Natural and Anthropogenic Drivers

**DOI:** 10.1371/journal.pone.0054260

**Published:** 2013-01-24

**Authors:** Ronaldo B. Francini-Filho, Ericka O. C. Coni, Pedro M. Meirelles, Gilberto M. Amado-Filho, Fabiano L. Thompson, Guilherme H. Pereira-Filho, Alex C. Bastos, Douglas P. Abrantes, Camilo M. Ferreira, Fernando Z. Gibran, Arthur Z. Güth, Paulo Y. G. Sumida, Nara L. Oliveira, Les Kaufman, Carolina V. Minte-Vera, Rodrigo L. Moura

**Affiliations:** 1 Departamento de Engenharia e Meio Ambiente, Universidade Federal da Paraíba, Rio Tinto, Paraíba, Brazil; 2 Departamento de Biologia, Universidade Estadual da Paraíba, Campina Grande, Paraíba, Brazil; 3 Departamento de Biologia Marinha, Universidade Federal do Rio de Janeiro, Rio de Janeiro, Brazil; 4 Instituto de Pesquisas Jardim Botânico do Rio de Janeiro, Rio de Janeiro, Brazil; 5 Departamento de Botânica, Universidade Federal Rural do Rio de Janeiro, Seropédica, Rio de Janeiro, Brazil; 6 Departamento de Oceanografia, Universidade Federal do Espírito Santo, Vitória, Espírito Santo, Brazil; 7 Centro de Ciências Naturais e Humanas, Universidade Federal do ABC, Santo André, São Paulo, Brazil; 8 Departamento de Oceanografia Biológica, Universidade de São Paulo, São Paulo, Brazil; 9 Departamento de Ciências Biológicas, Universidade Estadual de Santa Cruz, Ilhéus, Bahia, Brazil; 10 Boston University Marine Program, Boston, Massachusetts, United States of America; 11 Centro de Ciências Biológicas, Universidade Estadual de Maringá, Maringá, Paraná, Brazil; Consiglio Nazionale delle Ricerche (CNR), Italy

## Abstract

The Abrolhos Bank (eastern Brazil) encompasses the largest and richest coral reefs of the South Atlantic. Coral reef benthic assemblages of the region were monitored from 2003 to 2008. Two habitats (pinnacles' tops and walls) were sampled per site with 3–10 sites sampled within different reef areas. Different methodologies were applied in two distinct sampling periods: 2003–2005 and 2006–2008. Spatial coverage and taxonomic resolution were lower in the former than in the latter period. Benthic assemblages differed markedly in the smallest spatial scale, with greater differences recorded between habitats. Management regimes and biomass of fish functional groups (roving and territorial herbivores) had minor influences on benthic assemblages. These results suggest that local environmental factors such as light, depth and substrate inclination exert a stronger influence on the structure of benthic assemblages than protection from fishing. Reef walls of unprotected coastal reefs showed highest coral cover values, with a major contribution of *Montastraea cavernosa* (a sediment resistant species that may benefit from low light levels). An overall negative relationship between fleshy macroalgae and slow-growing reef-building organisms (i.e. scleractinians and crustose calcareous algae) was recorded, suggesting competition between these organisms. The opposite trend (i.e. positive relationships) was recorded for turf algae and the two reef-building organisms, suggesting beneficial interactions and/or co-occurrence mediated by unexplored factors. Turf algae cover increased across the region between 2006 and 2008, while scleractinian cover showed no change. The need of a continued and standardized monitoring program, aimed at understanding drivers of change in community patterns, as well as to subsidize sound adaptive conservation and management measures, is highlighted.

## Introduction

Worldwide, reef ecosystems are declining rapidly due to multiple disturbances such as climate change, overfishing, emerging diseases, pollution and sedimentation, implying severe losses of biodiversity and ecosystem services [1]–[3]. Despite the general consensus on the decline of coral reefs, there is little information on the dynamics of coral assemblages and the current status of reefs in large and important areas with tropical reefs, such as the South Atlantic [4]–[8].

The balance between abundance of relatively slow-growing reef building organisms (mainly crustose calcareous algae and scleractinian corals) and fast-growing non-building organisms (mainly turf and fleshy macroalgae) is one of the most widely used metric to evaluate reef condition, with dominance of the former indicating a healthy ecosystem [2], [3], [9]–[11]. Coral bleaching and disease, both triggered primarily by elevated sea surfaces temperatures, are main drivers of mass coral death [12]–[14]. Local anthropogenic disturbances, particularly nutrient overload and the overfishing of herbivores, lead to decreased reef resilience (i.e. lower capacity to recover after disturbance) and proliferation and persistence of algae after coral loss. Knowledge about major processes affecting such shifts from coral- to algal-dominated states (the so called “phase shifts”) is critical for the adequate conservation and management of coral reefs [3], [15]–[17].

The general perception of decline in coral cover is mostly based on long-term datasets and meta-analytical studies from the Caribbean and Indo-Pacific regions [2], [10]. However, even for these latter well studied regions, lack of historical baselines and long-term data obtained with standardized methodologies hampers accurate evaluations of reef conditions, leading to contrasting interpretations [11], [18], [19]. In addition, benthic coral reef assemblages vary greatly over several spatial and temporal scales, making it difficult to evaluate the relative importance of natural and anthropogenic drivers in assemblage patterns [20]–[22]. For example, high macroalgal cover may be determined by factors other than anthropogenic disturbances such as depth, nutrient and light availability, with shallow inshore sites generally showing higher fleshy macroalgal cover than deep offshore ones [23], [24].

Protection from fishing through establishment of no-take marine reserves may influence reef benthic assemblages via habitat protection and trophic cascading effects [25], [26]. For example, increased herbivory due to protection of populations of large roving reef fishes such as parrotfishes (Labridae) and surgeonfishes (Acanthuridae) may avoid macroalgal proliferation and thus facilitate coral recruitment and recovery after disturbance [25], [26]. Thus, it is not surprising that several studies have shown contrasting benthic communities between protected and unprotected reef sites [27], [28].

Brazilian coral reefs represent a priority area for biodiversity conservation in the Atlantic Ocean due to their relatively high endemism levels (about 25% for fishes and 30% for scleractinian corals) concentrated in a small reef area (5% of West Atlantic reefs) [6], [29]. Artisanal fisheries are largely unregulated and account for an estimated 70% of total fish landings in the Eastern Brazilian coast, where coral reefs are concentrated [4], [30]. Despite their importance, Brazilian reefs are under mounting anthropogenic disturbances, particularly overfishing, pollution and sedimentation [4], [31]–[33]. The recent proliferation of coral diseases in Brazil and the prognostic of mass death of a major endemic reef-building species (*Mussismilia braziliensis*) are of special concern [34].

This study aims to describe spatial and temporal patterns in reef benthic assemblages of the Abrolhos Bank, eastern Brazil, as well as to infer possible anthropogenic and natural processes/disturbances responsible for the observed patterns. The Abrolhos region encompasses the largest and richest coral reef complex in the South Atlantic Ocean and the oldest among the few networks of marine protected areas in the country [4].

## Materials and Methods

### Study region

The Abrolhos Bank (16°40′,19°40′S–39°10′, 37°20′W) is a wide portion of the continental shelf (46 000 km^2^), with depths rarely exceeding 30 m and a shelf edge at about 70 m. Reefs and rhodolith beds are the most prominent benthic features in the region [5]. Most reef structures display a characteristic form of mushroom-shaped pinnacles, which attain 5 to 25 m in height and 20 to 300 m across their tops [4]. Two main habitats can be distinguished in the reef pinnacles: tops (horizontal inclination; 2–6 m depth) and walls (vertical inclination; 3–15 m depth). About 20 scleractinian species are recorded for the region, at least six of them being endemic to Brazil [35].

Main rivers influencing the Abrolhos Bank are in its northern and southern extremes (River Jequitinhonha and River Doce, respectively). A large estuary formed by River Caravelas and River Peruípe is a remarkable feature of the coastline in the central portion of the Bank, near the main reefs [36]. Terrigenous sediments transported from land by river discharge predominate on coastal reefs, while biogenic carbonatic sediments predominate on mid- and outer-shelf reefs [37], [38]. Sedimentation regimes vary during the year, with higher sedimentation rates in winter-spring [33], [38], [39]. In summer, the rainfall is relatively high, leading to an increase in sediment transport to reefs via river discharge, while in winter resuspension of sediments is commonly caused by polar front winds [39].

### Sampling design and field measurements

**Figure 1:**
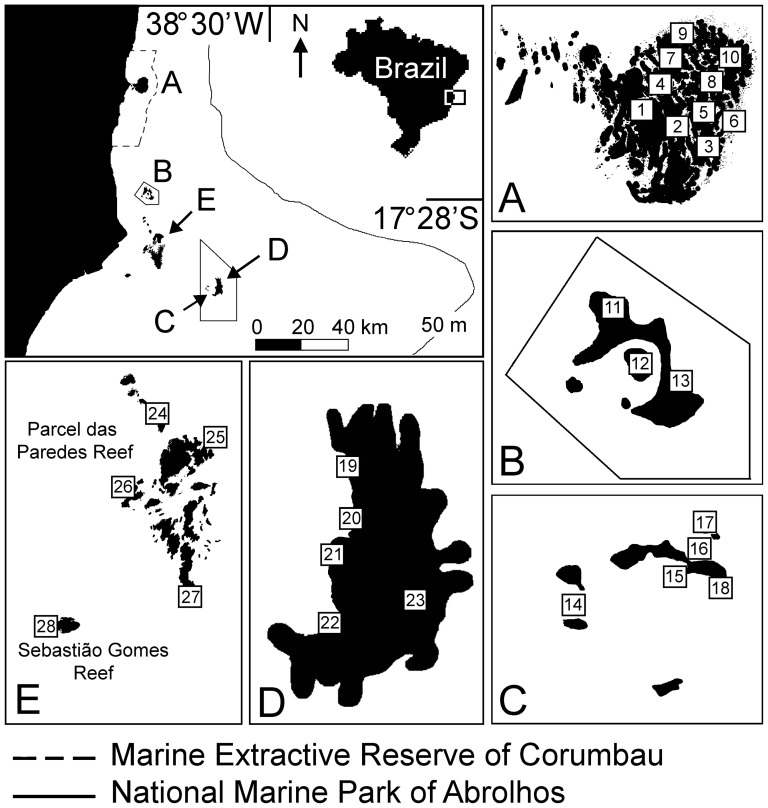
Map of the Abrolhos Bank, eastern Brazil, showing study sites and marine protected areas. A - Itacolomis Reef (no-take zone: sites 1–3; multiple-use zone: sites 4–10), B - Timbebas Reef, C - Abrolhos Archipelago, D - Parcel dos Abrolhos Reef, E – Unprotected coastal reefs.

The long-term monitoring program of coral reef benthic assemblages of the Abrolhos Bank started in 2003, through engagement of scientists and members of governmental and non-governmental organizations related to coastal management. Surveys were always carried out in the summer (January–March), thus avoiding seasonal artifacts. Each site was about 300 m in diameter and composed by 1–3 interconnected reef pinnacles, except for the rocky reefs of the Abrolhos Archipelago (see below). Spatial coverage and sampling methodologies varied through time, with two main periods. From 2003 to 2005 point-intercept lines (10 m length and 100 points; n = 4 per site) [40] were haphazardly placed on the pinnacle's tops, and groups of four quadrats (50×50 cm; 25 intercepts) equally distributed within 10 m lines were haphazardly placed on the pinnacle's walls. Each group of quadrats was considered as a single sample (n = 4 per site). Organisms immediately below each point were recorded *in situ* and classified as follows: turf algae, crustose calcareous algae, fire-corals (milleporids), fleshy macroalgae, live corals, octocorals and zoanthids. The “live coral” category includes only scleractinians, with no species distinction. During this first period, monitoring was performed in four areas (Fig. 1), as follows: **Area 1) No-take reserve of Timbebas Reef** (three sampling sites) – Located within the National Marine Park of Abrolhos (NMPA). Created by the Brazilian government in 1983, the NMPA comprises two discontinuous portions, one closer to the coast and poorly enforced (Timbebas Reef), and another farther from the coast and more intensively enforced (Abrolhos Archipelago and Parcel dos Abrolhos Reef). **Areas 2 and 3) Multiple-use and no-take zones of Itacolomis Reef –** Itacolomis Reef is the largest reef complex (∼50 km^2^) within the Marine Extractive Reserve of Corumbau (MERC) [41], [42]. It is divided into two main zones: multiple-use (Area 2; seven sampling sites) and no-take (Area 3; three sampling sites). **Area 4) Unprotected coastal reefs** (five sampling sites) – It encompasses the Parcel das Paredes Reef and Sebastião Gomes Reef, both subjected to the highest fishing pressure in the region [4] (Fig. 1).

Between 2006 and 2008 benthic assemblages were characterized using fixed photo-quadrats [34] in both, reef tops and walls (n = 10 per site). Each sample was composed by a mosaic of 15 high-resolution digital images totaling 0.7 m^2^. Quadrats were permanently delimited by fixed metal pins and set at haphazardly distances along 20–50 m axes. Relative cover of different benthic organisms was estimated through the identification of organisms (lowest taxonomic level possible) below 300 randomly distributed points per quadrat (i.e., 20 points per photograph) using the Coral Point Count with Excel Extensions Software [43]. Besides sampling the same sites within the abovementioned areas, two additional areas were sampled between 2006 and 2008, the Abrolhos Archipelago (five sampling sites) and Parcel dos Abrolhos Reef (five sampling sites), both within the NMPA portion that is farther from the coast (Fig. 1). The Abrolhos Archipelago is a rocky reef with no clear distinction between reef tops and walls, thus a single habitat (the reef front) was sampled. In total, 27 sites were sampled and 448 photo-quadrats were obtained per year between 2006 and 2008. A summary of the environmental characteristics of each sampling site is shown in Table S1.

Logistical support and research permits were provided by Parque Nacional Marinho de Abrolhos and Reserva Extrativista Marinha de Corumbau/ICMBio (through J.R.S. Neto, R. Jerolisky and R. Oliveira). Data from this work was made available for public access through the Dryad platform (http://datadryad.org/).

### Data analyses

Detailed analyses were performed for the period between 2006 and 2008 (“short-term comparisons”), in which data was obtained with a higher taxonomic resolution and a greater spatial coverage (see above). Inferences for the entire sampling period (2003–2008, “long-term comparisons”) were performed by making separate analyses for the two sampling periods: 2003–2005 and 2006–2008, and by considering only the same sampling sites and benthic categories (i.e. by standardizing data obtained in the two sampling periods). Long-term changes were taken into account only when similar trends were recorded for both sampling periods.

Some metal pins marking the fixed photo-quadrats were lost during the sampling period. These samples were excluded from the analyses in order to assure that exactly the same photo-quadrats were used for the temporal comparisons. Final sample size ranged between 7–10 quadrats per habitat per site per year. Three common genera of fleshy macroalgae (*Canistrocarpus* spp., *Dictyota* spp. and *Dictyopteris* spp.) were difficult to distinguish in the images, thus being pooled into a single category (hereafter called “other fleshy macroalgae”). All scleractinians were identified to the species level, except for *Siderastrea* spp., a genus for which three morphologically similar species are recorded for Brazil (*S. stellata*, *S. siderea* and *S. radians*) [44]. Data was also pooled for two morphologically similar fire-coral species (*Millepora brasiliensis* and *M. alcicornis*), but treated separately for the small-sized and conspicuous *Millepora nitida*.

Analysis of variance (ANOVA) was used to evaluate spatial and temporal variations in benthic cover. Two separate groups of ANOVA were calculated, the first one focusing on differences between tops and walls (considering reef pinnacles only) and the second one focusing on differences between reefs while ignoring between-habitat variability, this latter including the shallow rocky reefs of the Abrolhos Archipelago (which has no distinction between tops and walls). Because data could not be collected in the tops of three reefs (see Table S1), between-site variability was ignored in the ANOVA models, thus avoiding missing observations and the need of application of a less robust ANOVA model. In order to satisfy ANOVA assumptions of normality and homocedasticity, benthic cover percentages were converted to arcsin √x. Student-Newman-Keuls (SNK) multiple comparisons of means were performed as a *post-hoc* test [45].

Non-metric multidimensional scaling (MDS) ordination was used to summarize spatial and temporal similarities (Bray-Curtis) on the structure of benthic assemblages, and separate one-way analyses of similarities (ANOSIM) were used to evaluate significant differences according to reef areas, habitats and years [46].

Canonical correspondence analysis [47] was used to evaluate the influence of ecological and environmental explanatory variables on the structure (i.e. composition and relative cover) of benthic assemblages. Three fish functional groups are likely to exert strong influence on the benthos: 1) Large-bodied scrapers and grazers (Labridae: Scarinae), 2) Large-bodied browsers (Labridae: Sparisomatinae) and 3) Small-bodied territorial damselfish (Pomacentridae) [24], [48]–[50]. Biomass estimates for these three functional groups, together with depth, latitude, distance offshore and levels of protection were used as explanatory variables in the canonical correspondence analysis. Data on fish biomass was obtained from previous surveys [4], [41]. A forward selection procedure was used to include only the most important independent variables in the model, i.e. those contributing to increase the explanatory power of the model. Only significant variables, as defined by a Monte Carlo permutation test (999 permutations), were included in the final model. Reef areas were dummy-coded for levels of protection from fishing, as follows: 1) open-access reefs, 2) Itacolomis Reef (multiple-use portion), 3) Itacolomis Reefs (young no-take reserve), 4) Timbebas Reef (old and poorly enforced no-take reserve) and 5) Abrolhos Archipelago and Parcel dos Abrolhos Reef (old and well enforced no-take reserve) (see [4] for detailed information on protection levels of these areas; see Table S1).

Multiple linear regression analyses [45] were used to evaluate the relative influence of major non-building organisms (i.e. turf algae, fleshy macroalgae and *Palythoa caribaeorum*) on the abundance of key reef-building organisms (scleractinians and crustose calcareous algae). Percentage cover data are compositional and thus subjected to constant sum constraint. Because this may mask true relationships among variables, analyses were performed using the centered log-ratio transformation [51].

## Results

### Short-term comparisons

**Figure 2:**
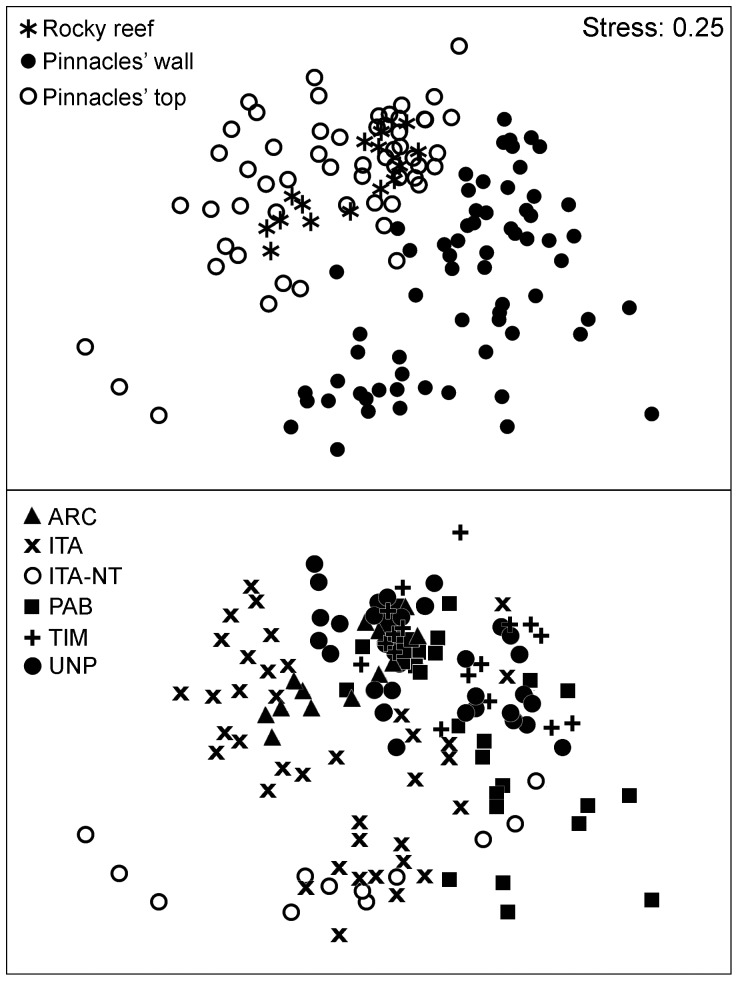
Multidimensional scaling (MDS) of benthic assemblages (i.e. relative cover of different organisms) based on Bray–Curtis similarities. Top panel: samples classified according to habitat; Bottom panel: samples classified according to reef areas. Reef areas: ARC – Archipelago, ITA-NT – Itacolomis Reef (no-take), ITA – Itacolomis Reef (multiple-use), PAB – Parcel dos Abrolhos (no-take), TIM – Timbebas Reef (no-take) and UNP – Unprotected coastal reefs.

**Figure 3:**
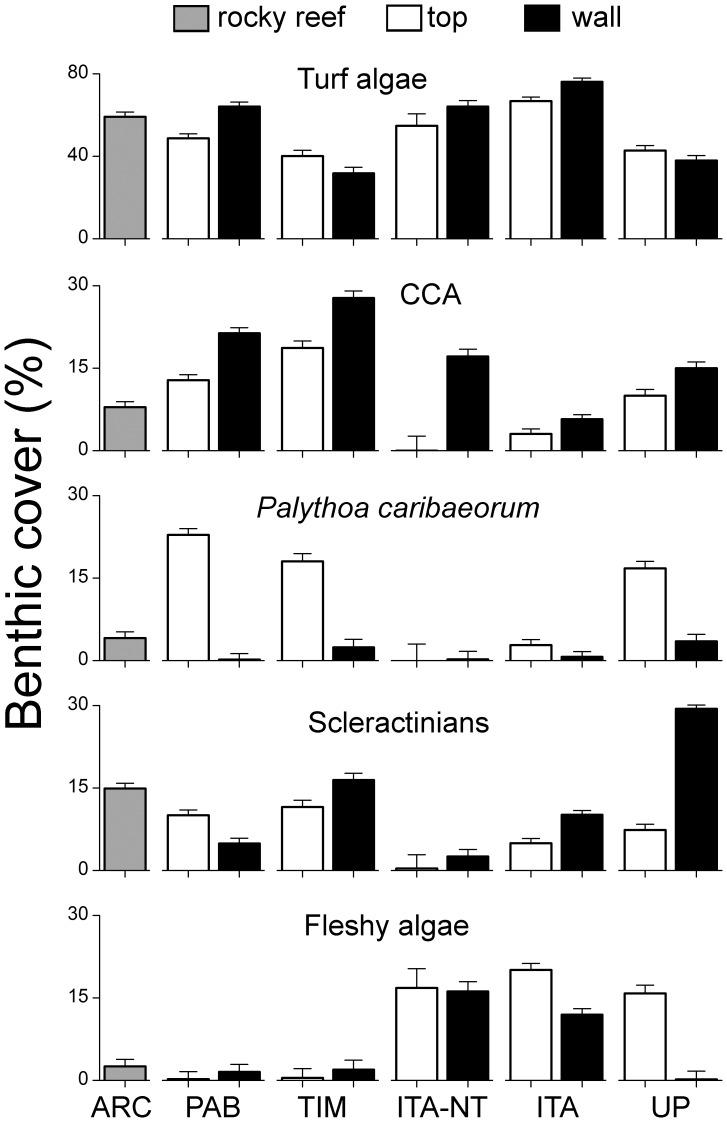
Benthic cover (mean + SE) of the top five most abundant organisms in the Abrolhos Bank.

**Figure 4:**
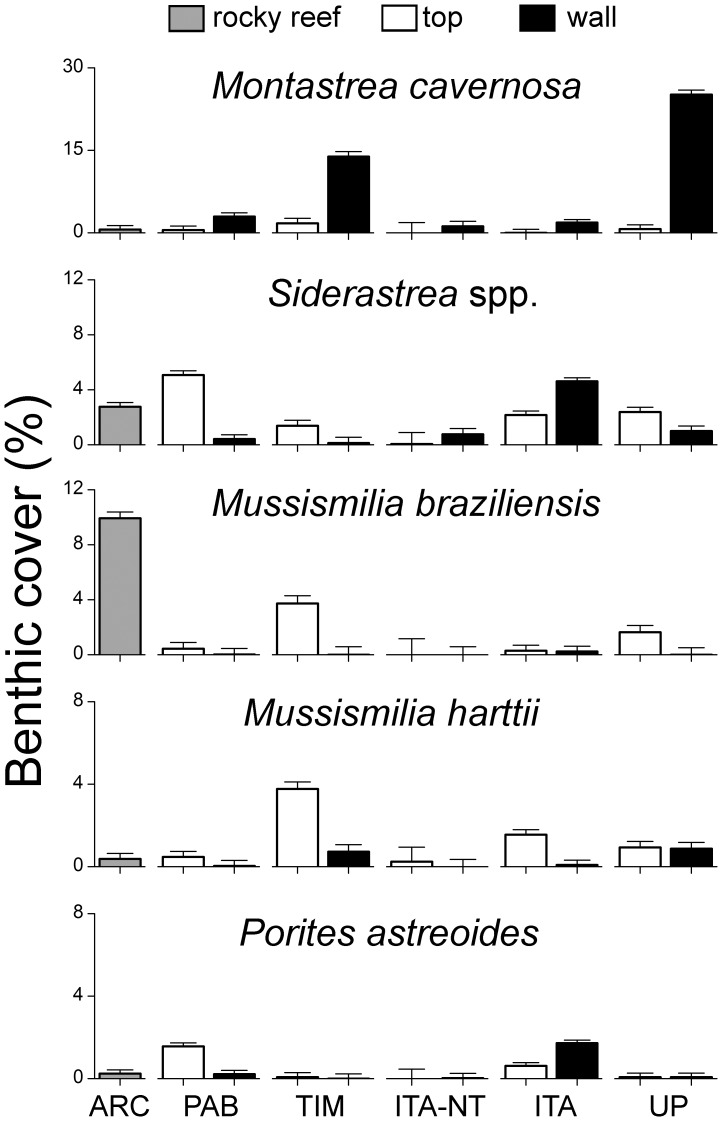
Benthic cover (mean + SE) of the top five most abundant reef corals (secleractinians) in the Abrolhos Bank.

The top five most abundant benthic organisms in the Abrolhos Bank, considering all sampling sites and years (2006–2008), belonged to different functional groups. Turf algae were by far the most abundant benthic organisms (56.1%), followed by crustose calcareous algae (12.1%), the zoanthid *Palythoa caribaeorum* (6.6%), the scleractinian coral *Monstastrea cavernosa* (4.1%) and the category composed by the fleshy macroalgae *Canistrocarpus* spp., *Dictyota* spp. and *Dictyopteris* spp. (2.9%) (Fig. S1). In ANOSIM analyses, values of global R were higher for contrasts between habitats (R = 0.26; P = 0.001) than reefs (R = 0.10; P = 0.003). The two-dimensional MDS ordination diagrams showed a much clearer distinction between habitats than between reefs, with samples from shallow rocky reefs of the Abrolhos Archipelago clustering together with samples from pinnacles' tops (Fig. 2). Benthic assemblages of both, pinnacles' tops and rocky reefs of the Abrolhos Archipelago, were characterized by relatively high covers of the scleractinian corals *Agaricia humilis*, *Favia gravida*, *Mussismilia braziliensis* and *Siderastrea* spp., articulated calcareous algae, as well as fleshy macroalgae of genus *Sargassum* (Figs. 3 and 4; Tables S2 and S3). Benthic assemblages of reef walls were characterized by high covers of the corals *Agaricia fragilis*, *Madracis decactis*, *M. cavernosa*, *Mussismilia hispida* and *Scolymia wellsi*, octocorals, sponges, ascidians, bryozoans, crustose calcareous algae, macroalgae of genus *Caulerpa* and cyanobacteria (Figs. 3 and 4; Tables S2 and S3).

Highest coral cover values were recorded at the walls of both protected (Timbebas Reef) and unprotected coastal reefs, with major contributions of *Montastraea cavernosa* and *Mussismilia hispida*. The reef coral *Mussismilia harttii*, octocorals, the algae *Halimeda* spp. and zoanthids of genus *Zoanthus* were also common at Timbebas, while the fire-coral *Millepora nitida* was also abundant at unprotected coastal reefs. The colonial zoanthid *P. caribaeorum* showed highest cover values at coastal reefs (both protected and unprotected) and at the mid-shelf fully-protected reefs of Parcel dos Abrolhos. Turf algae and fleshy macroalgae, particularly *Sargassum* spp., were more prevalent in the no-take and multiple-use zones of Itacolomis Reef (Figs. 3 and 4; Tables S2 and S3). Benthic assemblages of the Abrolhos Archipelago were dominated by the reef corals *Favia gravida*, *M. braziliensis* and *Siderastrea* spp., articulated calcareous algae and *Sargassum* spp. (Figs. 3 and 4; Table S4).

**Figure 5:**
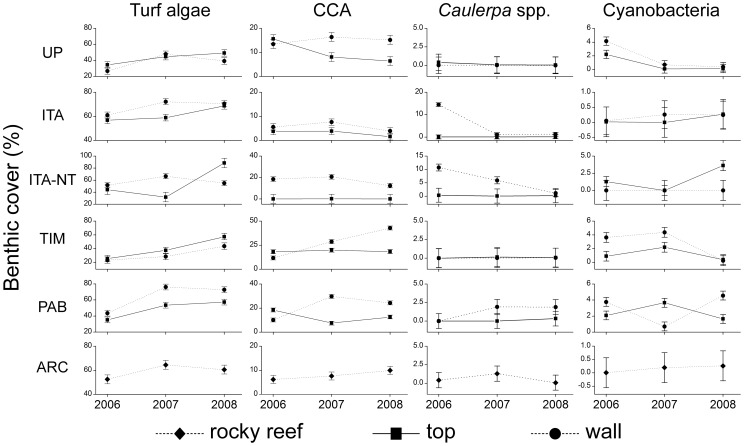
Temporal dynamics in cover (mean ± SE) of benthic organisms in the Abrolhos Bank between 2006 and 2008. Only organisms for which significant temporal variations were recorded are shown.

No significant between-years variation was detected on the structure of benthic assemblages (R = 0.008; P = 0.8). However, significant variations were recorded for several individual organisms/categories. Most noticeable was the increase in turf algae cover in most reef areas between 2006 and 2008 (Fig. 5; Tables S2 and S3). Crustose calcareous algae declined sharply on reef tops of unprotected coastal reefs. Temporal dynamics of *Caulerpa* spp. and cyanobacteria was not consistent among reef areas, with significant interactions being recorded (Fig. 5; Tables S2 and S3).

The multiple regression models explained a much higher variance for scleractinian corals (r^2^ = 0.57) than for crustose calcareous algae (r^2^ = 0.13). Negative relationships were recorded between fleshy macroalgae and reef-building organisms, the opposite trend (i.e. positive relationships) being recorded between turf algae and reef-building organisms. The zoanthid *P. caribaeorum* showed a negative effect on the cover of crustose calcareous algae and a weak yet significant positive effect on the cover of scleractinian corals (Table S5).

**Figure 6:**
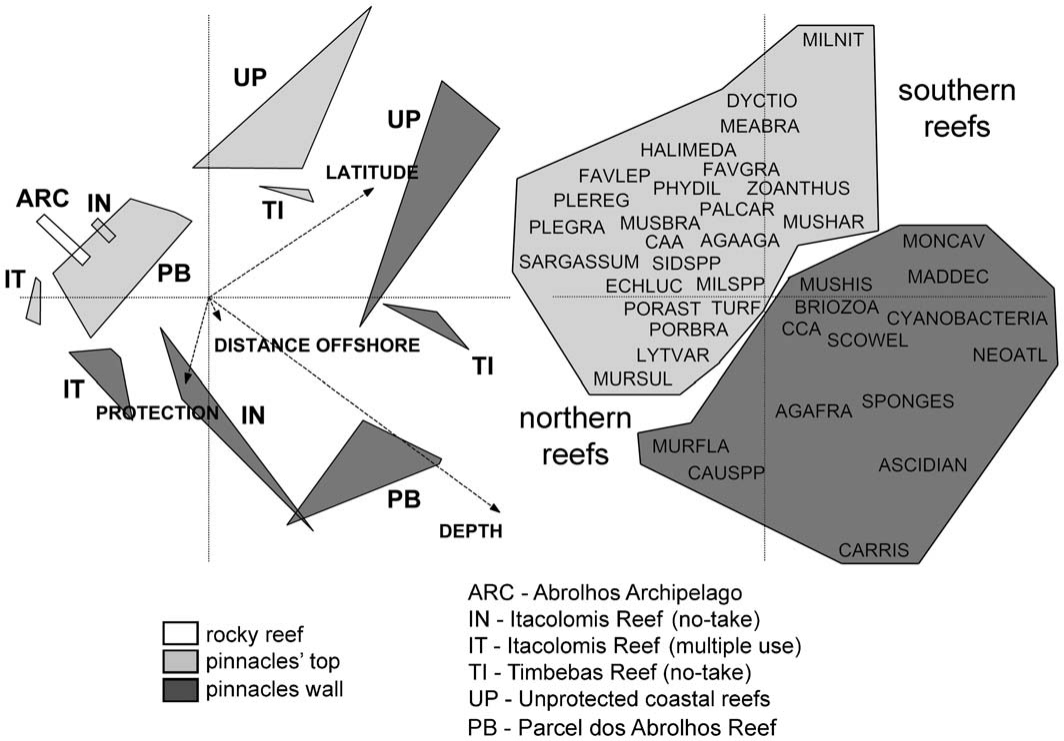
Canonical correspondence analysis plot showing: (left) relationship between independent variables (arrows) and reef areas and (right) distribution of benthic organisms in the two-dimensional ordination space.

Depth, latitude, distance offshore and protection levels were, in decreasing order (i.e. order of entrance in the model), the four most important variables affecting the structure of benthic assemblages. Latitude and distance offshore were positively correlated with each other, with southern reefs (Parcel dos Abrolhos Reef) more distant from the coast than northern ones (Itacolomis Reef), making difficult to disentangle the effect of these two explanatory variables. The first two axes explained 75.3% of the relationship between environmental characteristics and benthic assemblages' structure. Inclusion of the remaining three explanatory variables (i.e. biomass of scrapers, grazers and territorial herbivores) increased the power of explanation of the model by less than 1%. Most importantly, these latter three variables were not significant (P>0.05) according to the Monte Carlo test. There was a clear distinction of samples obtained in different habitats and reef areas in the two-dimensional ordination diagram (Fig. 6). Four main reef benthic assemblages were recorded: 1) Reef tops of northern/inshore reefs, 2) Reef tops of southern/offshore reefs, 3) Reef walls of northern/inshore reefs and 4) Reef walls of southern/offshore reefs (Fig. 6).

### Long-term comparisons

**Figure 7:**
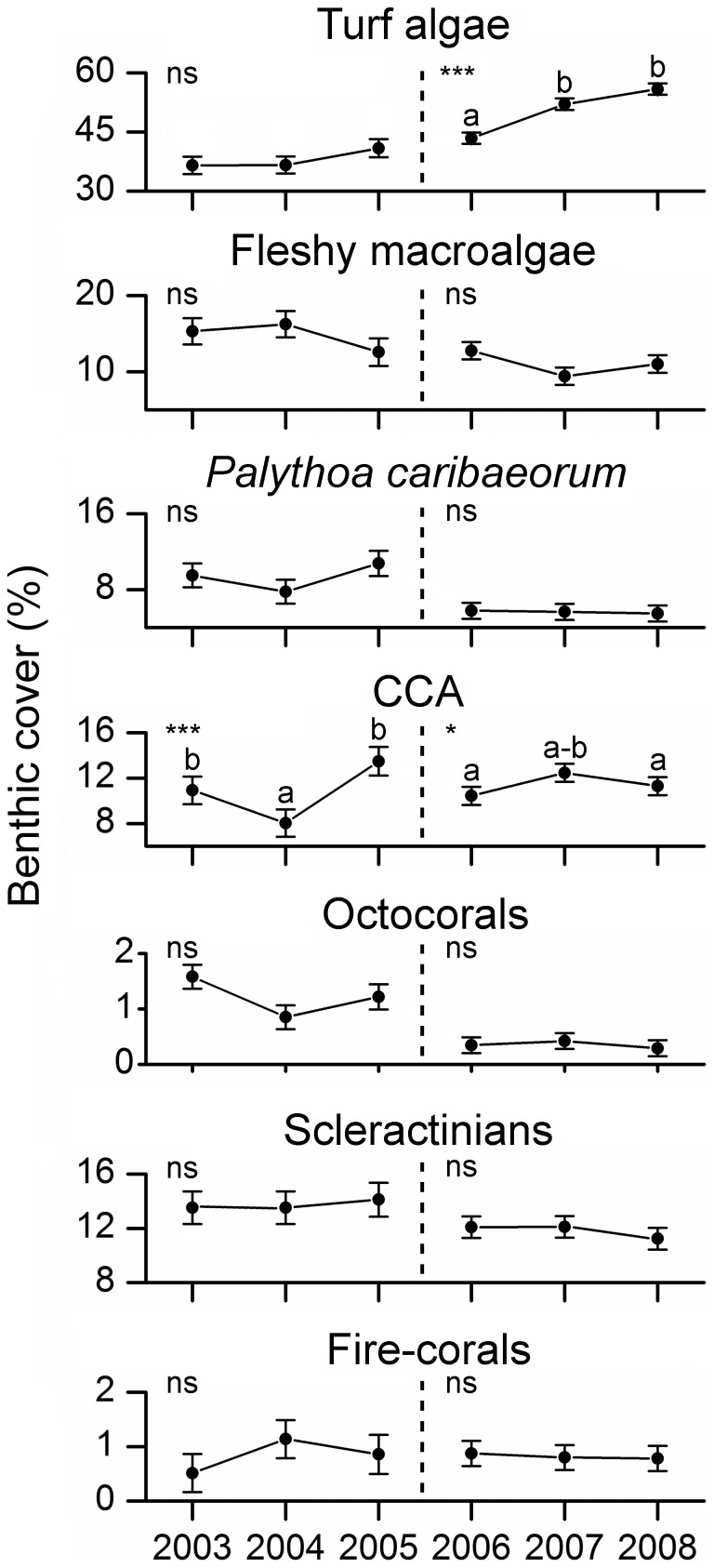
Temporal dynamics in cover (mean ± SE) of benthic organisms in the Abrolhos Bank between 2003 and 2008. The dashed line separates the two sampling periods in which different methodologies were used (see Materials and Methods). Analyses of Variance (ANOVA) results: *P<0.05, **P<0.01, ***P<0.001. Homogeneous groups are identified by equal letters.

**Figure 8:**
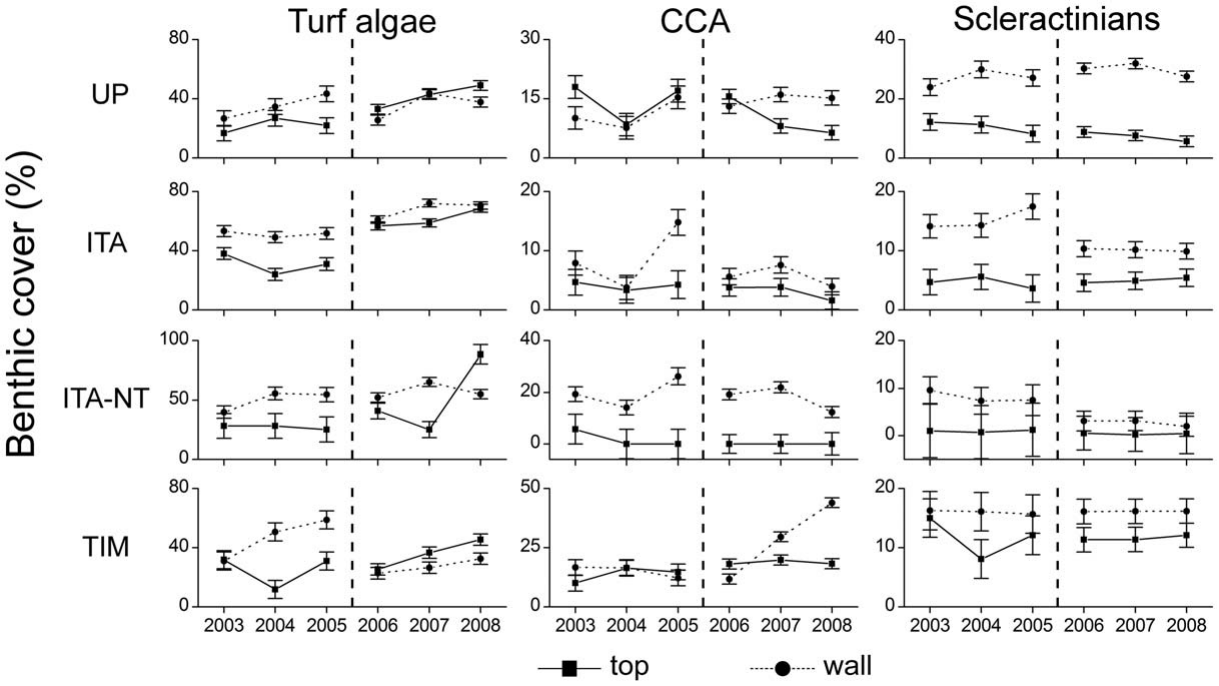
Temporal dynamics in cover (mean ± SE) of benthic organisms in the Abrolhos Bank between 2003 and 2008 considering different reef areas and habitats. Reef areas: ITA-NT – Itacolomis Reef (no-take), ITA – Itacolomis Reef (multiple-use), TIM – Timbebas Reef (no-take) and UNP – Unprotected coastal reefs. The dashed line separates the two sampling periods in which different methodologies were used (see Materials and Methods).

**Figure 9:**
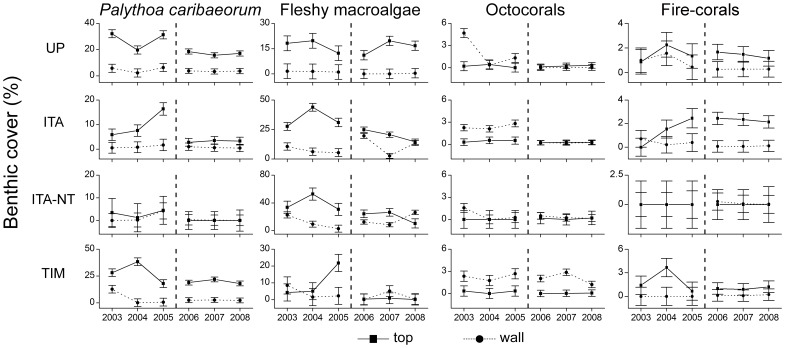
Temporal dynamics in cover (mean ± SE) of benthic organisms in the Abrolhos Bank between 2003 and 2008 considering different reef areas and habitats. Reef areas: ITA-NT – Itacolomis Reef (no-take), ITA – Itacolomis Reef (multiple-use), TIM – Timbebas Reef (no-take) and UNP – Unprotected coastal reefs. The dashed line separates the two sampling periods in which different methodologies were used (see Materials and Methods).

When considering data pooled for all habitats and sites, temporal variations between 2003 and 2008 (i.e. for both sampling periods) were recorded for CCA only, with no clear overall trend of increase or decrease through time (Fig. 7). A significant increase in turf algae cover was recorded for the entire Abrolhos Bank in the second period of the study (2006–2008) (Fig. 7). All interactions were significant in this latter case, with increases recorded for most habitats/reefs, except for reef walls of the no-take zone of Itacolomis Reef (Fig. 8; Table S6). Octocoral cover declined on reef tops of the unprotected coastal reefs between 2003 and 2005 (Fig. 9; Table S6). No other significant temporal variations were recorded (Figs. 7, 8 and 9; Table S6).

## Discussion

This is one of the most comprehensive characterizations of shallow coral reef benthic assemblages of the Abrolhos Bank performed to date and one of the few studies focusing on a relatively long temporal scale [8], [52]. The first study focusing on coral cover in the region was performed by [53]. Unfortunately, direct comparisons between data from [53] and data obtained here are not possible, as in the former study no absolute coral cover values were given, only species percentages in relation to total coral cover. Other relevant studies in the Abrolhos Bank were performed with no clear specification of site location [54], by using different methodologies [55], [56] and/or by applying different metrics [52], [55]. Thus, caution is needed when performing between-studies comparisons (see below).

**Figure 10:**
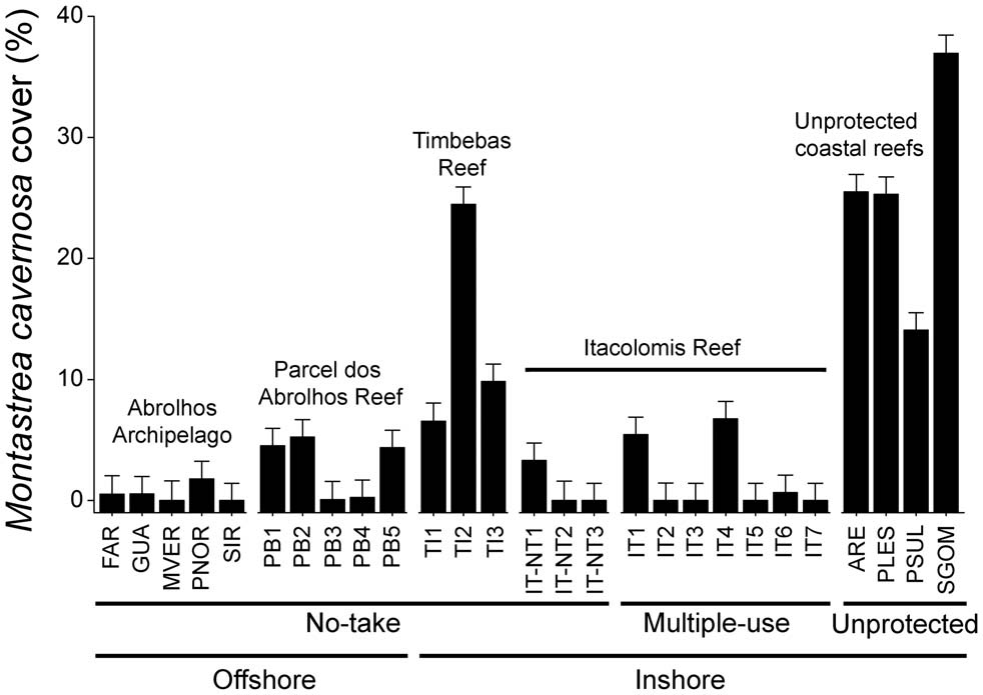
Cover (mean + SE) of the reef coral *Montastraea cavernosa* in reef walls of the Abrolhos Bank (data pooled for samples obtained between 2006 and 2008).

In the present study, highest coral cover values (with a major contribution of *Montastraea cavernosa*) were recorded in reef walls of unprotected coastal reefs (Fig. 10). It is important to note that most previous studies focusing on coral reef benthic assemblages of the Abrolhos Bank have sampled reef tops only, but see [52], thus underestimating the relevance of coastal reefs in terms of coral cover [8], [33], [53], [54]. For example, [8] stated that “Because the lateral walls of these pinnacles are mostly inhabited by small coral colonies (such as *Agaricia fragilis*, *Scolymia wellsi*, *Meandrina braziliensis*) that do not have great importance as reef builders, they were not assessed”. This limitation in the sampling design of previous studies is particularly important considering the results obtained here showing that differences between habitats are more important than differences between reefs. Results from this study indicate that *M. cavernosa* is a major reef-building species in SW Atlantic coral reefs, highlighting the importance of studies focusing particularly on the healthy and dynamics of this species. In the Caribbean, habitats dominated by *Montastraea* spp. have the highest biodiversity and support the largest number of ecosystem processes and services [57]. Other scleractinians characteristics of reef walls commonly recorded here (*Agaricia fragilis*, *Mussismilia hispida* and *Scolymia wellsi*) may have also been underestimated in previous studies, but see [52]. Only one previous study conducted in the Abrolhos region has also sampled reef tops and wall [52]. Results from this latter study (based on semi-quantitative and presence-absence data obtained in 42 sites), also indicated significant differences in the structure of coral assemblages (hidrocorals, octocorals, scleractinians and *P. caribaeorum*) between tops and walls.

The high coverage of *M. cavernosa* on walls of inshore unprotected reefs is not surprising. *Montastraea cavernosa* is recognized as a sediment resistant species with a high capacity for sediment removal, being a major component of the “sediment resistant coral fauna” in the West Atlantic [58]. *Montastraea cavernosa* is also abundant in sediment-free mesophotic (30–150 m) reef communities [59], [60], which suggests that this species benefits from low light levels. The same pattern (i.e. occurrence in shallow turbid reefs and mesophotic clear water reefs) was recently recorded for *A. fragilis*, *M. hispida* and *S. wellsi* [60]. Another possible explanation for the high coverage of *M. cavernosa* on inshore reefs of the Abrolhos Bank is the relatively high availability of nutrients in inshore reefs, which may lead to high coral growth rates and reproductive output [61], [62].

The strong between-habitat differences recorded here suggest that factors such as light, depth and bottom inclination are the main drivers of benthic assemblages' structure in the Abrolhos region. Other studies performed elsewhere have highlighted the importance of such environmental variables for coral reef benthic assemblages [63]–[65]. Due to the peculiar growth form of reef pinnacles in the Abrolhos region, depth, inclination and light levels vary sharply in a scale of just a few meters, accounting for the extreme variability recorded here. In this study, only weak relationships were recorded between protection levels and the structure of benthic assemblages. In particular, no relationship between biomass of herbivorous fish and the structure of benthic assemblages were recorded. Thus, although previous studies have shown that protection afforded by no-take reserves within the Abrolhos Bank lead to increased fish biomass, including large herbivorous fish [4], [24], [41], it is suggested here that fish recoveries are still incipient to promote noticeable changes in benthic assemblages [66], [67]. Depending on the degree of reef degradation, up to 10 years of effective protection may be necessary for detecting changes in benthic assemblages [68]. The Brazilian-endemic brain coral *Mussismilia braziliensis* was an exception to the abovementioned pattern, as this species was relatively more abundant inside no-take zones (Timbebas Reefs and the Abrolhos Archipelago) (see Fig. 4). [52] also recorded higher cover values of *M. braziliensis* inside no-take zones within the Abrolhos Bank (Parcel dos Abrolhos Reef and Timbebas Reefs). A recent study [28] has shown that no-take zones of the Abrolhos Bank may promote coral reef health, with noticeable positive effects on microbial, benthic and fish assemblages. However, the sampling design of this latter study was limited to the spatial comparison of reef tops of four sampling sites only, with no formal evaluation of the relative influence of different factors (e.g. habitat characteristics and protection levels).

The relatively high cover of the zoanthid *Palythoa caribaeorum* in the Abrolhos Bank is noteworthy (up to 25% in some sites). This species is an aggressive competitor for space, killing or inhibiting the growth of nearly all other sessile reef invertebrates, including corals [69], thus playing important roles in reef community processes [70]. The negative relationship between cover of *P. caribaeorum* and that of crustose calcareous algae suggests competition between these organisms. [38] sampled three sites also included in this study (Pedra de Leste, Ponta Sul and Parcel dos Abrolhos) and found a negative relationship between distance offshore and *P. caribaeorum* cover, the opposite trend being recorded for scleractinians. Such a pattern was not recorded here, with highest values of *P. caribaeorum* cover recorded in tops of both inshore unprotected coastal reefs and tops of mid-shelf fully-protected reefs (Parcel dos Abrolhos Reef). [38] suggested that sedimentation levels may mediate competition between *P. caribaeorum* and scleractinians, with high sedimentation levels favoring the former. Sedimentation, desiccation and predation levels are some of the environmental and ecological drivers that may influence *P. caribaeorum* abundance [70]–[72], but detailed experimental studies are still needed in order to understand factors affecting abundance and competitive capabilities of *P. caribaeorum* in the Abrolhos Bank and elsewhere.

A moderate increase in turf algae cover was recorded across the Abrolhos Bank between 2006 and 2008. The lack of relationship between biomass of herbivorous fish and algae abundance reported here indicate that other factors, such as coral mortality *per se* due to diseases [34] and nutrient enrichment [28] are more important than herbivory levels for controlling turf algae abundance in the Abrolhos Bank. The lack of temporal variation for most benthic organisms/categories recorded here suggests that longer-term data may be necessary in order to detect possible shifts in coral reef benthic assemblages of the Abrolhos region and to better understand the underlying processes.

Results from the multiple regression models obtained here indicate that competition with fleshy macroalgae is important for both scleractinian corals and crustose calcareous algae. Negative effects of fleshy macroalgae on corals have been widely reported [73]–[75]. The strong positive relationship between turf algae cover and scleractinian cover recorded here was surprising, as turf algae may also cause deleterious effects to scleractinians [76]–[78]. However, some coral species may suffer no effects or even be competitively superior to turf algae. The variation in the outcomes of interactions between scleractinians and turf algae may be related to several factors. For example, relatively large and massive coral colonies may be competitively superior to turf algae than small branching ones [79], [80]. Algal identity is also important, with different species showing negative or null effects on scleractinians [81]. In some particular cases, turf algae may exert positive effects by providing species-specific settlement cues for scleractinians [82].

The lack of historical ecological data impedes the understanding of processes underlying community-level dynamics and the evaluation of the actual degree of conservation/degradation of reef communities [11], [83]. In this regard, large spatial and temporal scale monitoring programs such as the present one may provide key data for understanding drivers of change in community patterns and for creating sound adaptive conservation and management measures [83], [84]. As more long-term monitoring results are made available, more comprehensive qualitative [85] and meta-analytical studies using data from different geographical regions will be made possible [2], [10], [84]. Data from Brazil may be of particular interest when testing hypotheses related to the effects of functional diversity on assemblage resistance/resilience, given the low species richness and functional redundancy of Brazilian reefs.

## Supporting Information

Figure S1 **Decreasing order of abundance of benthic organisms in the Abrolhos Bank.** Species codes: First three letters of genus name followed by first three letters of specific epithet (see full names in Table S2).
(TIF)

Table S1 **Summary of environmental characteristics of sampling reefs and sites.**
(DOC)

Table S2 **Analyses of Variance (ANOVA) testing the effect of reef areas (R), habitats (H) and years (Y) in cover of different benthic organisms with data obtained between 2006 and 2008.** The Abrolhos Archipelago area (rocky reef) was excluded from these analyses in order to allow a more comprehensive comparison between pinnacles' tops and walls (see Material and Methods).
(DOC)

Table S3 **Significant differences in benthic cover according to reef areas (R), habitats (H) and years (Y), as determined by Student-Newman-Keuls (SNK)** ***post-hoc*** **comparisons.** Reefs arranged in decreasing order of benthic cover, with homogeneous groups linked by an equal sign. Reef areas: IN – Itacolomis Reef (no-take), IT – Itacolomis Reef (multiple-use), PB – Parcel dos Abrolhos (no-take), TI – Timbebas Reef (no-take), UP – Unprotected coastal reefs. Habitats: TP – tops and WA – walls. Years: 2006–2008. The Abrolhos Archipelago area (shallow rocky reef) was excluded from these analyses in order to allow a more comprehensive comparison between pinnacles' tops and walls (see Material and Methods).
(DOC)

Table S4 **Analyses of Variance (ANOVA) testing the effect of reef areas (R) and years (Y) in cover of different benthic organisms with data obtained between 2006 and 2008.** Reef areas: AR – Abrolhos Archipelago, IN – Itacolomis Reef (no-take), IT – Itacolomis Reef (multiple-use), PB – Parcel dos Abrolhos (no-take), TI – Timbebas Reef (no-take), UP – Unprotected coastal reefs.
(DOC)

Table S5 **Multiple regression results showing the relative influence of fast growing non-reef building organisms (turf alga, fleshy algae and** ***Palythoa caribaeorum*** **) on abundance of key reef-building organisms (scleractinians and crustose calcareous algae).** Levels of significance for full model and partial *r^2^*: * P<0.05; ** P<0.01; *** P<0.001.
(DOC)

Table S6 **Analyses of Variance (ANOVA) testing the effect of reef areas, habitats and years in cover of different benthic organisms for the two sampling periods (2003–2005/2005–2008).**
(DOC)
