# Supplementary figures and images for: Dynamics of Coral Reef Benthic Assemblages of the Abrolhos Bank, Eastern Brazil: Inferences on Natural and Anthropogenic Drivers
Source: PLoS One. 2013 Jan 24;8(1):e54260. doi: 10.1371/journal.pone.0054260 (PMC3554776; doi:10.1371/journal.pone.0054260)

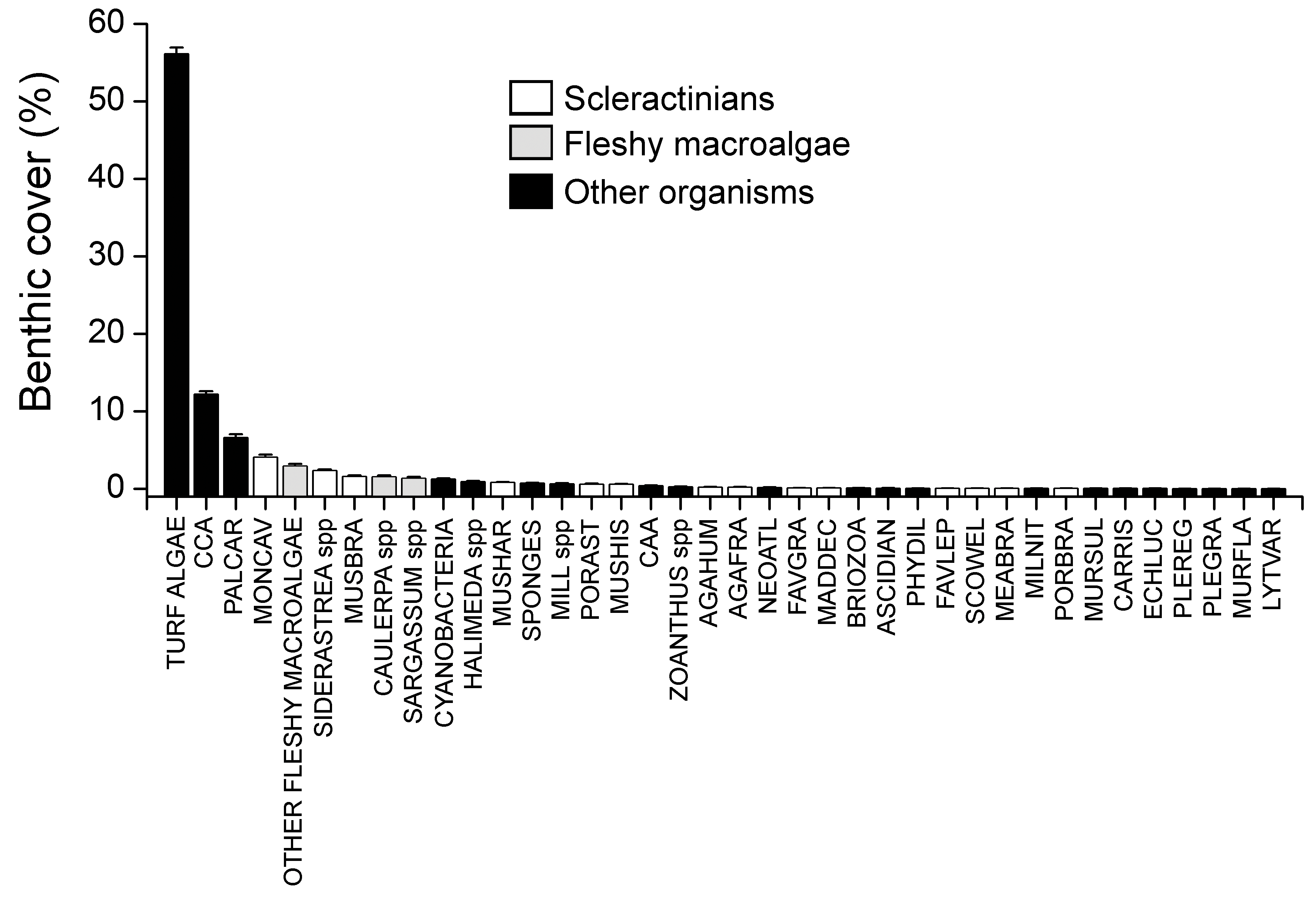

Supplement: Figure S1 — Decreasing order of abundance of benthic organisms in the Abrolhos Bank. Species codes: First three letters of genus name followed by first three letters of specific epithet (see full names in Table S2). (TIF) [file pone.0054260.s001.tif]
