# Supplementary material for: Dynamics of Coral Reef Benthic Assemblages of the Abrolhos Bank, Eastern Brazil: Inferences on Natural and Anthropogenic Drivers
Source: PLoS One. 2013 Jan 24;8(1):e54260. doi: 10.1371/journal.pone.0054260 (PMC3554776; doi:10.1371/journal.pone.0054260)
Supplement: Table S1 — Summary of environmental characteristics of sampling reefs and sites. (DOC) [file pone.0054260.s002.doc]

Table S1

| Reef area / Sampling site | Habitat / Depth (m) | | Distance offshore  (km) | | | Protection level  (dummy-coded) | | Geographic position ^a^  (lat / long) | |  |
| --- | --- | --- | --- | --- | --- | --- | --- | --- | --- | --- |
| Abrolhos Archipelago | Reef front | |  | | |  | |  | |  |
| Farol (FAR) | 5.8 | | 58.1 | | | 5 | | -17.965 / -38.694 | |  |
| Guarita (GUA) | 5.4 | | 58.1 | | | 5 | | -17.960 / -38.692 | |  |
| Mato Verde (MVER) | 4.1 | | 58.1 | | | 5 | | -17.964 / -38.702 | |  |
| Portinho Norte (PNOR) | 5.4 | | 58.1 | | | 5 | | -17.959 / -38.701 | |  |
| Siriba (SIR) | 3.1 | | 58.1 | | | 5 | | -17.968 / -38.707 | |  |
| Itacolomis Reef (multiple-use) | | Top | | Wall |  | |  | |  | |
| ITA1 | | 1.3 | | 4.2 | 5.5 | | 2 | | -16.899 / -39.063 | |
| ITA2 | | 2.2 | | 6.2 | 7.9 | | 2 | | -16.905 / -39.041 | |
| ITA3 | | - | | 12.3 | 8.5 | | 2 | | -16.903 / -39.031 | |
| ITA4 | | 1.5 | | 4.5 | 5.2 | | 2 | | -16.896 / -39.063 | |
| ITA5 | | 1.5 | | 3.6 | 7.5 | | 2 | | -16.901 / -39.042 | |
| ITA6 | | 1.4 | | 4.3 | 5.2 | | 2 | | -16.892 / -39.063 | |
| ITA7 | | 2.6 | | 5.6 | 7.8 | | 2 | | -16.898 / -39.040 | |
| Itacolomis Reefs (no-take) | | Top | | Wall |  | |  | |  | |
| ITA-NT1 | | 1.7 | | 4.6 | 5.8 | | 3 | | -16.909 / -39.060 | |
| ITA-NT2 | | - | | 6.7 | 8.3 | | 3 | | -16.912 / -39.038 | |
| ITA-NT3 | | - | | 11.9 | 9.0 | | 3 | | -16.914 / -39.030 | |
| Parcel dos Abrolhos Reef | | Top | | Wall |  | |  | |  | |
| PAB1 | | 5.9 | | 14.7 | 63.4 | | 5 | | -17.991 / -38.650 | |
| PAB2 | | 7.3 | | 16.9 | 61.4 | | 5 | | -17.983 / -38.667 | |
| PAB3 | | 6.9 | | 17.0 | 61.7 | | 5 | | -17.998 / -38.671 | |
| PAB4 | | 7.7 | | 15.2 | 61.7 | | 5 | | -17.959 / -38.655 | |
| PAB5 | | 5.6 | | 15.0 | 60.1 | | 5 | | -17.944 / -38.659 | |
| Timbebas Reef | |  | |  |  | |  | |  | |
| TIM1 | | 4.7 | | 9.5 | 19.0 | | 4 | | -17.482 / -39.013 | |
| TIM2 | | 6.0 | | 11.9 | 17.4 | | 4 | | -17.477 / -39.027 | |
| TIM3 | | 5.6 | | 10.5 | 17.0 | | 4 | | -17.460 / -39.031 | |
| Unprotected Coastal Reefs | | Top | | Wall |  | |  | |  | |
| Arenguera (AREN) | | 5.2 | | 8.8 | 15.0 | | 1 | | -17.666 / -38.997 | |
| Ponta Sul (PSUL) | | 4.4 | | 10.8 | 31.4 | | 1 | | -17.880 / -38.938 | |
| Pedra de Leste (PLES) | | 2.3 | | 6.1 | 14.3 | | 1 | | -17.783 / -39.051 | |
| Sebastião Gomes (SG) | | 3.2 | | 8.2 | 13.7 | | 1 | | -17.913 / -39.145 | |

^a^Datum: WGS84
