# Supplementary material for: Dynamics of Coral Reef Benthic Assemblages of the Abrolhos Bank, Eastern Brazil: Inferences on Natural and Anthropogenic Drivers
Source: PLoS One. 2013 Jan 24;8(1):e54260. doi: 10.1371/journal.pone.0054260 (PMC3554776; doi:10.1371/journal.pone.0054260)
Supplement: Table S2 — Analyses of Variance (ANOVA) testing the effect of reef areas (R), habitats (H) and years (Y) in cover of different benthic organisms with data obtained between 2006 and 2008. The Abrolhos Archipelago area (rocky reef) was excluded from these analyses in order to allow a more comprehensive comparison between pinnacles' tops and walls (see Material and Methods). (DOC) [file pone.0054260.s003.doc]

Table S2

|  | R | H | Y | R x H | R x Y | H x Y | R x H x Y |
| --- | --- | --- | --- | --- | --- | --- | --- |
| Fire-corals |  |  |  |  |  |  |  |
| *Millepora nitida* | *** | *** | ns | *** | ns | ns | ns |
| *Millepora* spp.^a^ | ** | *** | ns | ns | ns | ns | ns |
| Total fire-corals | *** | ** | ns | ** | ns | ns | ns |
| Scleractinians |  |  |  |  |  |  |  |
| *Agaricia fragilis* | *** | *** | ns | *** | ns | ns | * |
| *Agaricia humilis* | *** | *** | ns | *** | *** | ns | *** |
| *Favia gravid* | *** | *** | ns | ** | * | ns | ns |
| *Favia leptophylla* | ns | * | ns | ns | ns | ns | ns |
| *Madracis decactis* | ** | *** | ns | ** | ns | ns | ns |
| *Meandrina braziliensis* | * | ns | ns | ns | * | ns | * |
| *Montastraea cavernosa* | *** | *** | ns | *** | ns | ns | ns |
| *Mussismilia braziliensis* | *** | *** | ns | *** | ns | ns | ns |
| *Mussismilia hartti* | *** | *** | ns | ** | ns | ns | ns |
| *Mussismilia hispida* | *** | *** | ns | *** | ns | ns | ns |
| *Porites astreoides* | *** | ns | ns | *** | ns | ns | ns |
| *Porites branneri* | ns | ns | ns | ns | ns | ns | ns |
| *Scolymia wellsi* | ns | *** | ns | ns | ns | ns | ns |
| *Siderastrea* spp. | *** | *** | ns | *** | ns | ns | ns |
| Total scleractinians | *** | *** | ns | *** | ns | ns | ns |
| Octocorals^b^ | *** | * | ns | *** | ns | ns | ns |
| Sea urchins^c^ | *** | * | ns | *** | ns | ns | ns |
| Sponges | *** | *** | ** | *** | ** | ns | * |
| Ascidians | *** | *** | ns | *** | *** | ns | ** |
| Bryozoans | *** | ** | ns | ** | *** | ns | ns |
| Crustose calcareous algae (CCA) | *** | *** | ** | *** | *** | *** | *** |
| Calcareous articulated algae (CAA) | *** | *** | *** | *** | *** | ** | *** |
| *Halimeda* spp. | *** | *** | ns | *** | ns | ns | ns |
| Cyanobacteria | *** | *** | * | ** | *** | * | *** |
| Turf algae | *** | ns | *** | *** | ** | *** | * |
| Fleshy macroalgae |  |  |  |  |  |  |  |
| *Caulerpa* spp. | *** | *** | * | *** | *** | * | *** |
| *Sargassum* spp. | *** | *** | ns | *** | ns | ns | ns |
| Other fleshy macroalgae^d^ | *** | *** | ns | *** | ** | * | ** |
| Total fleshy macroalgae | *** | *** | ns | *** | *** | * | ** |
| Zoanthids |  |  |  |  |  |  |  |
| *Palythoa caribaeorum* | *** | *** | ns | *** | ns | ns | ns |
| *Zoanthus* spp. | *** | *** | * | *** | *** | ns | ns |
| Total zoanthids | *** | *** | ns | *** | ns | ns | ns |

*P < 0.05, **P < 0.01, ***P < 0.001, ns - not significant;

^a^Data pooled for *Millepora alcicornis* and *M. brasiliensis*

^b^Data pooled for *Carijoa riisei*, *Muriceopsis sulphurea*, *Muricia flama*, *Neospongodes atlantica*, *Phyllogorgia dilatata*, *Plexaurella grandiflora* and *Plexaurella regia*

^c^Data pooled for *Echinometra lucunter* and *Lytechinus variegatus*

^d^Data pooled for *Canistrocarpus* spp*. + Dictyopteris* spp. + *Dictyota* spp.
