# Supplementary material for: Dynamics of Coral Reef Benthic Assemblages of the Abrolhos Bank, Eastern Brazil: Inferences on Natural and Anthropogenic Drivers
Source: PLoS One. 2013 Jan 24;8(1):e54260. doi: 10.1371/journal.pone.0054260 (PMC3554776; doi:10.1371/journal.pone.0054260)
Supplement: Table S3 — Significant differences in benthic cover according to reef areas (R), habitats (H) and years (Y), as determined by Student-Newman-Keuls (SNK) post-hoc comparisons. Reefs arranged in decreasing order of benthic cover, with homogeneous groups linked by an equal sign. Reef areas: IN – Itacolomis Reef (no-take), IT – Itacolomis Reef (multiple-use), PB – Parcel dos Abrolhos (no-take), TI – Timbebas Reef (no-take), UP – Unprotected coastal reefs. Habitats: TP – tops and WA – walls. Years: 2006–2008. The Abrolhos Archipelago area (shallow rocky reef) was excluded from these analyses in order to allow a more comprehensive comparison between pinnacles' tops and walls (see Material and Methods). (DOC) [file pone.0054260.s004.doc]

Table S3

|  | R | H | Y |
| --- | --- | --- | --- |
| Fire-corals |  |  |  |
| *Millepora nítida* | UP > TI = PB = IT = IN | TP > WA | ns |
| *Millepora* spp. | UP = TI = IT = PB > IT = PB = IN | TP > WA | ns |
| Total fire-corals | UP = TI = IT > TI = IT = PB > IT = PB = IN | TP > WA | ns |
| Scleractinians |  |  |  |
| *Agaricia fragilis* | PB > IT > UP = IN = TI | WA > TP | ns |
| *Agaricia humilis* | PB > UP = TI = IT > IT = IN | TP > WA | ns |
| *Favia gravid* | TI = UP > UP = PB = IT > IN | TP > WA | ns |
| *Favia leptophylla* | Ns | TP > WA | ns |
| *Madracis decactis* | UP = TI = PB > TI = PB = IT = IN | WA > TP | ns |
| *Meandrina braziliensis* | UP > PB = IT = TI = IN | ns | ns |
| *Montastraea cavernosa* | UP > TI > PB = IT = IN | WA > TP | ns |
| *Mussismilia braziliensis* | TI > UP > IT = PB = IN | TP > WA | ns |
| *Mussismilia hartti* | TI > UP > IT = PB > PB = IN | TP > WA | ns |
| *Mussismilia hispida* | UP > TI > IT = PB = IN | WA > TP | ns |
| *Porites astreoides* | IT = PB > UP = TI = IN | ns | ns |
| *Porites branneri* | Ns | ns | ns |
| *Scolymia wellsi* | Ns | WA > TP | ns |
| *Siderastrea* spp. | IT > PB > UP > TI = IN | TP > WA | ns |
| Total scleractinians | UP > TI > IT = PB > IN | WA > TP | ns |
| Octocorals^a^ | TI > IT = IN = UP > IN = UP = PB | WA > TP | ns |
| Sea urchins^b^ | IT > UP = TI = IN = PB | TP > WA | ns |
| Sponges | PB = UP > TI > IT = IN | WA > TP | ns |
| Ascidians | PB > IN = TI = UP = IT | WA > TP | ns |
| Bryozoans | UP = PB > IT = IN = TI | WA > TP | ns |
| Crustose calcareous algae | TI > PB > UP > IN > IT | WA > TP | 07 = 08 > 08 = 06 |
| Calcareous articulated algae | TI = IN = PB > UP > IT | TP > WA | 07 > 06 = 08 |
| *Halimeda* spp. | TI > UP = IN > IN = IT > PB | TP > WA | ns |
| Cyanobacteria | PB = TI > UP = IN > IT | WA > TP | 06 = 07 > 07 = 08 |
| Turf algae | IT > IN > PB > UP > TI | ns | 08 > 07 > 06 |
| Fleshy macroalgae |  |  |  |
| *Caulerpa* spp. | IN > IT > PB = UP = TI | WA > TP | 06 > 07 = 08 |
| *Sargassum* spp. | IT > IN = TI = UP = PB | TP > WA | ns |
| Other fleshy macroalgae^c^ | UP = IN > IN = IT > TI = PB | TP > WA | ns |
| Total macroalgae | IN = IT > UP > TI = PB | TP > WA | ns |
| Zoanthids |  |  |  |
| *Palythoa caribaeorum* | TI = PB = UP > IT = IN | TP > WA | ns |
| *Zoanthus* spp. | TI > UP = PB = IT > IN | TP > WA | 07 > 06 = 08 |
| Total zoanthids | TI = UP = PB > IT > IN | TP > WA | ns |

^a^Data pooled for *Millepora alcicornis* and *M. brasiliensis*

^b^Data pooled for *Carijoa riisei*, *Muriceopsis sulphurea*, *Muricia flama*, *Neospongodes atlantica*, *Phyllogorgia dilatata*, *Plexaurella grandiflora* and *Plexaurella regia*

^c^Data pooled for *Echinometra lucunter* and *Lytechinus variegatus*

^d^Data pooled for *Canistrocarpus* spp*. + Dictyopteris* spp. + *Dictyota* spp.
