# Supplementary material for: Dynamics of Coral Reef Benthic Assemblages of the Abrolhos Bank, Eastern Brazil: Inferences on Natural and Anthropogenic Drivers
Source: PLoS One. 2013 Jan 24;8(1):e54260. doi: 10.1371/journal.pone.0054260 (PMC3554776; doi:10.1371/journal.pone.0054260)
Supplement: Table S4 — Analyses of Variance (ANOVA) testing the effect of reef areas (R) and years (Y) in cover of different benthic organisms with data obtained between 2006 and 2008. Reef areas: AR – Abrolhos Archipelago, IN – Itacolomis Reef (no-take), IT – Itacolomis Reef (multiple-use), PB – Parcel dos Abrolhos (no-take), TI – Timbebas Reef (no-take), UP – Unprotected coastal reefs. (DOC) [file pone.0054260.s005.doc]

Table S4

|  | R | | Y | | | R x Y | | | | *Post-hoc* |  | | |
| --- | --- | --- | --- | --- | --- | --- | --- | --- | --- | --- | --- | --- | --- |
|  | |  | |  | | | |  | R | | | | Y |
| Fire-corals | |  | |  |  | |  | | | | |  | |
| *Millepora nitida* | | *** | | ns | ns | | UP > AR = TI = PB = IT = IN | | | | | ― | |
| *Millepora* spp. | | * | | ns | ns | | UP = TI = AR = IT = PB > IT = PB = IN | | | | | ― | |
| Total fire-corals | | *** | | ns | ns | | UP = TI = AR = IT > TI = AR = IT = PB > IT = PB = IN | | | | | ― | |
| Scleractinians | |  | |  |  | |  | | | | |  | |
| *Agaricia fragilis* | | *** | | ns | ns | | PB .> IT = UP = IN > UP = IN = TI = AR | | | | | ― | |
| *Agaricia humilis* | | *** | | ns | *** | | PB > AR = UP = TI = IT > IT = IN | | | | | ― | |
| *Favia grávida* | | *** | | ns | ns | | AR > TI = UP = PB > UP = PB = IT > IN | | | | | ― | |
| *Favia leptophylla* | | ** | | ns | ns | | AR > TI = PB = UP = IT = IN | | | | | ― | |
| *Madracis decactis* | | *** | | ns | ns | | UP = TI = PB > TI = PB = IT = IN = AR | | | | | ― | |
| *Meandrina braziliensis* | | * | | ns | * | | UP = PB > IT = AR = TI = IN | | | | | ― | |
| *Montastraea cavernosa* | | *** | | ns | ns | | UP > TI > UPB = AR = IT = IN | | | | | ― | |
| *Mussismilia braziliensis* | | *** | | ns | ns | | AR > TI > UP = IT = PB > IT = PB = IN | | | | | ― | |
| *Mussismilia hartti* | | *** | | ns | ns | | TI > UP > IT = AR = PB > PB = IN | | | | | ― | |
| *Mussismilia hispida* | | *** | | ns | ns | | UP > TI > AR = IT = PB = IN | | | | | ― | |
| *Porites astreoides* | | *** | | ns | ns | | IT = PB > AR = UP = TI = IN | | | | | ― | |
| *Porites branneri* | | ns | | ns | ns | | ― | | | | | ― | |
| *Scolymia wellsi* | | ** | | ns | ns | | UP = TI = IT = PB > TI = IT = PB = IN > PB = IN = AR | | | | | ― | |
| *Siderastrea* spp. | | *** | | ns | ns | | AR > IT > PB > UP > TI = IN | | | | | ― | |
| Total scleractinians | | *** | | ns | ns | | UP = AR > AR = TI > IT = PB > IN | | | | | ― | |
| Octocorals^a^ | | *** | | ns | ns | | TI > IT = IN = UP > IN = UP = AR = PB | | | | | ― | |
| Sea urchins^b^ | | *** | | ns | ns | | IT > AR = UP = TI = IN = PB | | | | | ― | |
| Sponges | | *** | | * | ** | | PB = UP > UP = TI > AR = IT > IT = IN | | | | | 08 > 07 = 06 | |
| Ascidians | | *** | | ns | *** | | PB > IN = TI = UP = IT = AR | | | | | ― | |
| Bryozoans | | *** | | * | *** | | UP = PB > AR = IT = IN = TI | | | | | 07 = 08 > 06 | |
| Crustose calcareous algae | | *** | | ** | *** | | TI > PB > UP = IN > IN = AR > IT | | | | | 07 = 08 > 06 | |
| Calcareous articulated algae | | *** | | *** | *** | | AR > TI = IN = PB = UP = IT | | | | | 07 = 06 > 08 | |
| *Halimeda* spp. | | *** | | * | ns | | TI > AR = UP > UP = IN = IT > IN = IT = PB | | | | | ― | |
| Cyanobacteria | | *** | | ns | *** | | PB = TI > UP = IN > AR = IT | | | | | ― | |
| Turf algae | | *** | | *** | * | | IT > AR = IN > PB > UP > TI | | | | | 08 > 07 > 06 | |
| Fleshy macroalgae | |  | |  |  | |  | | | | |  | |
| *Caulerpa* spp. | | *** | | *** | *** | | IN = IT > PB = AR = UP = TI | | | | | 06 > 07 = 08 | |
| *Sargassum* spp. | | *** | | ns | ns | | AR = IT > IN = TI = UP = PB | | | | | ― | |
| Other fleshy macroalgae^c^ | | *** | | ns | ns | | UP = IN > ITA-NT = IT = AR > TI = PB | | | | | ― | |
| Total macroalgae | | *** | | ns | *** | | IN = IT > AR = UP > TI = PB | | | | | ― | |
| Zoanthids | |  | |  |  | |  | | | | |  | |
| *Palythoa caribaeorum* | | *** | | ns | ns | | TI = PB = UP > AR > IT = IN | | | | | ― | |
| *Zoanthus* spp. | | *** | | * | *** | | TI > AR = UP = PB = IT > IN | | | | | 07 > 06 = 08 | |
| Total zoanthids | | *** | | ns | ns | | TI = PB = UP > AR > IT = IN | | | | | ― | |

*P < 0.05, **P < 0.01, ***P < 0.001, ns - not significant;

^a^Data pooled for *Millepora alcicornis* and *M. brasiliensis*

^b^Data pooled for *Carijoa riisei*, *Muriceopsis sulphurea*, *Muricia flama*, *Neospongodes atlantica*, *Phyllogorgia dilatata*, *Plexaurella grandiflora* and *Plexaurella regia*

^c^Data pooled for *Echinometra lucunter* and *Lytechinus variegatus*

^d^Data pooled for *Canistrocarpus* spp*. + Dictyopteris* spp. + *Dictyota* spp.
