# Supplementary material for: Dynamics of Coral Reef Benthic Assemblages of the Abrolhos Bank, Eastern Brazil: Inferences on Natural and Anthropogenic Drivers
Source: PLoS One. 2013 Jan 24;8(1):e54260. doi: 10.1371/journal.pone.0054260 (PMC3554776; doi:10.1371/journal.pone.0054260)
Supplement: Table S5 — Multiple regression results showing the relative influence of fast growing non-reef building organisms (turf alga, fleshy algae and Palythoa caribaeorum ) on abundance of key reef-building organisms (scleractinians and crustose calcareous algae). Levels of significance for full model and partial r2: * P<0.05; ** P<0.01; *** P<0.001. (DOC) [file pone.0054260.s006.doc]

Table S5

| Reef building organisms | *r^2^* | *P* | | | Partial *r^2^* | | |  | | | | | |
| --- | --- | --- | --- | --- | --- | --- | --- | --- | --- | --- | --- | --- | --- |
|  | | |  | | |  | | | Turf algae | | Fleshy algae | | *P. caribaeorum* |
| Scleractinians | | | 0.57 | *** | | | 0.81*** | | | -0,29*** | | 0.18** | |
| Crustose calcareous algae | | | 0.13 | *** | | | 0.24** | | | -0.33*** | | -0.23* | |
