# Supplementary material for: Dynamics of Coral Reef Benthic Assemblages of the Abrolhos Bank, Eastern Brazil: Inferences on Natural and Anthropogenic Drivers
Source: PLoS One. 2013 Jan 24;8(1):e54260. doi: 10.1371/journal.pone.0054260 (PMC3554776; doi:10.1371/journal.pone.0054260)
Supplement: Table S6 — Analyses of Variance (ANOVA) testing the effect of reef areas, habitats and years in cover of different benthic organisms for the two sampling periods (2003–2005/2005–2008). (DOC) [file pone.0054260.s007.doc]

Table S6

| Sampling period / benthic category | R | H | Y | R x H | R x Y | H x Y | R x H x Y |
| --- | --- | --- | --- | --- | --- | --- | --- |
| 2003-2005 |  |  |  |  |  |  |  |
| Crustose calcareous algae | *** | ns | ** | *** | ns | ns | ns |
| Fire-corals | *** | * | ns | ns | ns | ns | ns |
| Fleshy macroalgae | *** | *** | ns | *** | ns | ** | ns |
| Octocorals | ns | *** | ** | ns | * | ** | * |
| *Palythoa caribaeorum* | *** | *** | ns | *** | ** | ns | ** |
| Scleractinians | *** | *** | ns | *** | ns | ns | ns |
| Turf algae | *** | *** | ** | * | *** | * | *** |
| 2006-2008 |  |  |  |  |  |  |  |
| Crustose calcareous algae | *** | *** | ** | *** | *** | *** | *** |
| Fire-corals | *** | ** | ns | ** | ns | ns | ns |
| Fleshy macroalgae | *** | *** | ns | *** | *** | * | ** |
| Octocorals | *** | * | ns | *** | ns | ns | ns |
| *Palythoa caribaeorum* | *** | *** | ns | *** | ns | ns | ns |
| Scleractinians | *** | *** | ns | *** | ns | ns | ns |
| Turf algae | *** | ns | *** | *** | ** | *** | * |

*P < 0.05, **P < 0.01, ***P < 0.001, ns - not significant
